# Zinc-Modified Titanate Nanotubes as Radiosensitizers for Glioblastoma: Enhancing Radiotherapy Efficacy and Monte Carlo Simulations

**DOI:** 10.1021/acsomega.4c02125

**Published:** 2024-06-28

**Authors:** Fernando Mendonça Diz, Wesley F. Monteiro, Iury Santos Silveira, Daniel Ruano, Eduardo Rosa Zotti, Rafael Diogo Weimer, Micael Nunes Melo, João Gabriel Schossler Lopes, Thamiris Becker Scheffel, Linda V. E. Caldas, Jaderson Costa da Costa, Fernanda Bueno Morrone, Rosane Angélica Ligabue

**Affiliations:** † Preclinical Research Center, Brain Institute of Rio Grande do Sul, Pontifical Catholic University of Rio Grande do Sul—PUCRS, Porto Alegre, Rio Grande do Sul 90619-900, Brazil; ‡ Graduate Program in Materials Engineering and Technology, Pontifical Catholic University of Rio Grande do Sul—PUCRS, Porto Alegre, Rio Grande do Sul 90619-900, Brazil; § Institute of Energy and Nuclear Research, National Nuclear Energy Commission—IPEN/CNEN. São Paulo, São Paulo 01151, Brazil; ∥ ALBA Syconhrotron Light Source, Cerdanuola del Vallès 08290, Spain; ⊥ Instituto de Tecnología Química, Universitat Politècnica de València-Consejo Superior de Investigaciones Científica (UPV-CSIC), Valencia 46022, Spain; # Institute of Technology and Research—ITP, Aracaju, Sergipe 49032-490 Brazil; ¶ Radiotherapy Service at Hospital São Lucas da Pontifical Catholic University of Rio Grande do Sul/Oncoclinic Group, Porto Alegre, Rio Grande do Sul 90619-900, Brazil; ∇ School of Life and Health Sciences, Pontifical Catholic University of Rio Grande do Sul—PUCRS, Porto Alegre, Rio Grande do Sul 90619-900, Brazil

## Abstract

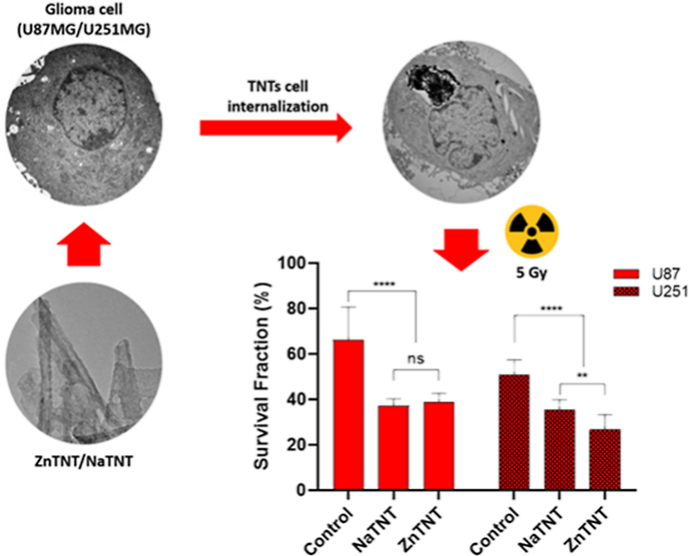

Radiotherapy (RT) is the established noninvasive treatment for glioblastoma (GBM), a highly aggressive malignancy. However, its effectiveness in improving patient survival remains limited due to the radioresistant nature of GBM. Metal-based nanostructures have emerged as promising strategies to enhance RT efficacy. Among them, titanate nanotubes (TNTs) have gained significant attention due to their biocompatibility and cost-effectiveness. This study aimed to synthesize zinc-modified TNTs (ZnTNT) from sodium TNTs (NaTNT), in addition to characterizing the formed nanostructures and evaluating their radiosensitization effects in GBM cells (U87 and U251). Hydrothermal synthesis was employed to fabricate the TNTs, which were characterized using various techniques, including transmission electron microscopy (TEM), energy-dispersive spectroscopy, scanning-transmission mode, Fourier-transform infrared spectroscopy, ICP-MS (inductively coupled plasma mass spectrometry), X-ray photoelectron spectroscopy, and zeta potential analysis. Cytotoxicity was evaluated in healthy (Vero) and GBM (U87 and U251) cells by the MTT assay, while the internalization of TNTs was observed through TEM imaging and ICP-MS. The radiosensitivity of ZnTNT and NaTNT combined with 5 Gy was evaluated using clonogenic assays. Monte Carlo simulations using the MCNP6.2 code were performed to determine the deposited dose in the culture medium for RT scenarios involving TNT clusters and cells. The results demonstrated differences in the dose deposition values between the scenarios with and without TNTs. The study revealed that ZnTNT interfered with clonogenic integrity, suggesting its potential as a powerful tool for GBM treatment.

## 1 Introduction

Radiotherapy (RT) is a therapeutic modality that involves the transfer and deposition of high-energy radiation to treat several types of solid tumors.^1−3^ The primary target of ionizing radiation is DNA, which can be directly or indirectly affected, leading to the generation of free radicals and subsequent cell death.^4,5^ However, exposure to ionizing radiation can cause damage to both tumor and normal cells.^6,7^ To minimize harm to normal tissues, the radiation dose administered during therapy is limited, which can compromise the effectiveness of tumor cell eradication.^8,9^ In recent decades, nanomedicine has garnered significant attention as a potential strategy to improve the efficiency of radiation deposition to tumor tissue while minimizing adverse effects on healthy tissue caused by conventional cancer RT.^10,11^

In the current clinical scenario, the use of metallic nanoparticles is already a reality, and metal-based NPs have been approved for some medical applications (cancer RT, contrast agent, and iron replacement therapy).^12^ In RT specifically, nanotechnology employing metal nanoparticles has been studied extensively as a new approach for the diagnosis and treatment of malignant tumors due to their unique physicochemical and biological properties.^13,14^ Recently, hafnium oxide nanoparticles, HfO_2_NPs (marketed as NBTXR3), have been approved to improve radiosensitization in patients with soft tissue sarcoma.^11,15^ While HfO_2_NPs were the first metal-based nanoparticles used for the treatment of solid tumors, further technological advancements are needed to enhance the field of nanomedicine in clinical cancer RT.^16^

Titanate nanotubes (TNTs) have demonstrated promise as radiosensitizers in preclinical trials for cancer RT, as they possess the ability to enhance the effectiveness of X-rays and γ-rays.^17,18^ The tubular morphology of TNTs is an important feature enabling their cellular internalization through mechanisms such as endocytosis and diffusion without causing cytotoxicity.^19,20^ Additionally, hydrothermally synthesized TNTs can be easily modified with various inorganic and organic compounds, which increase radiation absorption,^21−25^ and facilitate the generation of reactive oxygen species (ROS).^26^

Given these properties, zinc (Zn) has emerged as a promising candidate for incorporating TNTs. Zinc has demonstrated a potential role in the prevention and treatment of several pathophysiological conditions, such as neurological diseases and cancer.^27−29^ In the field of biomedical applications, zinc-based materials offer attractive options due to their ability to modulate biocompatibility through factors such as shape, size, and Zn^2+^ concentration.^30,31^ Zinc has also been found to induce apoptosis in human cancer cells through ROS-mediated mechanisms.^32−34^ Furthermore, from a radiotherapeutic perspective, zinc exhibits radioluminescence, which enables the detection of ionizing energy.^35,36^

Glioblastoma (GBM; WHO grade IV astrocytoma) is the most common and fatal malignant primary brain tumor found in adults, with a median survival of 7–14 months.^37−39^ The standard treatment for GBM typically involves a multimodal approach consisting of surgical resection, adjuvant chemotherapy (Temozolomide), and RT.^40−42^ However, GBM cells are known to be radioresistant, necessitating the development of strategies to enhance tumor radiosensitivity and improve patient prognosis.^43^ Recent studies have focused on nanoscale devices to improve the efficacy of RT, leading researchers to propose new nanostructures (complex nanogold with outer-membrane vesicle; nanocarriers to improve delivery of anti-GBM drugs).^18,44−50^

As a way of understanding how ionizing radiation interacts with matter, some statistical methods are commonly used; the main one is the Monte Carlo Method (MCM). There are several tools that use MCM to simulate the radiation transport as MCNP, Fluka, Geant4, Penelope, and others.^51^ Monte Carlo N-Particle 6.2 (MCNP6.2) is the radiation transport code used to simulate the interaction of photon beams with TNTs and tumor tissue.^52^ MCNP version 6.2 allows tracking photons with energies up to 1 eV and electrons with an energy of 10 eV. These technical features enable studying the influence of small structures such as living cells and their surroundings.^53^ Furthermore, the results from the MCNP simulations show how the presence of TNTs influences the dose deposition in the cells and their surroundings.

Therefore, TNTs hold promise in this regard, as they can penetrate tumor cells and their external surface can be modified with various components to increase the absorption of ionizing radiation specifically in tumor cells. This work intends to contribute to the field of nanomedicine and potentially produce a significant advance in the treatment not only of GBM, but also of other radioresistant tumors. The goal of this work is to synthesize and characterize zinc-modified TNTs to enhance the radiosensitization effect in GBM cells as well as to determine the deposited dose using Monte Carlo simulations. Achieving this goal, this work aims to advance the field of nanomedicine and potentially yield a significant breakthrough in the treatment of not only GBM but also other radioresistant tumors.

## 2 Materials and Methods

### 2.1 Materials

Titanium dioxide (JB Qumica, TiO_2_, 98% anatase phase), sodium hydroxide (Vetec, 99%), zinc chloride (Vetec, 99%), titanium standard for ICP (Sigma-Aldrich), and dimethyl sulfoxide (DMSO) (Sigma-Aldrich, DMSO 99,5%) were used as received. The following materials were used for cellular management: Dulbecco’s Modified Eagle Medium (DMEM) media (DMEM), fetal bovine serum (FBS), penicillin–streptomycin (10,000 U/mL), and amphotericin B (Fungizone), which were obtained from Gibco, MTT (3-(4,5-dimethylthiazol-2-yl)-2,5-diphenyltetrazolium bromide solution—MTT 5 mg/mL in PBS in 90% culture medium supplemented with 10% FBS), Trypan Blue dye, Hoechst 33,342 (Sigma-Aldrich Canada), and calcium and magnesium-free medium (CMF).

### 2.2 Preparation of TNTs

#### 2.2.1 Synthesis of ZnTNT Nanostructures

Sodium TNTs (NaTNTs) were synthesized by the hydrothermal method as described in the literature.^54,55^ In a typical procedure, 1.5 g (18.7 mmol) of TiO_2_ was added to 120 mL of a 10 mol·L^–1^ NaOH solution. The suspension was placed under magnetic stirring at room temperature for 30 min. The suspension was then transferred to a stainless steel reactor (200 cm^3^) internally coated by Teflon maintained for 72 h at 135 °C. Next, a white precipitate was separated by centrifugation, washed with distilled water until pH = 8 (wash water), dried at 80 °C for 6 h, and kept in a desiccator. TNT synthesis with zinc (ZnTNT) was based on the method described by Monteiro et al.^54^ A typical procedure consisted of adding 1.0 g (3.3 mmol) of NaTNT to 100 mL of a 0.5 mol·L^–1^ ZnCl_2_ aqueous solution (50 mmol) under magnetic stirring for 15 min at room temperature. Next, the suspension was filtered under reduced pressure and washed with distilled water until complete chloride ion removal (silver nitrate test). The obtained white solid was dried at 80 °C for 6 h and kept in a desiccator.

#### 2.2.2 Characterization of ZnTNT Nanostructures

Transmission electron microscopy (TEM) of nanostructure samples was realized using copper grids with carbon film (300 mesh) in FEI Tecnai G2 T20 equipment. TNT dimensions were obtained by the TEM images using *ImageJ* software (number of measurements *n* = 25). Nanostructure mapping by energy-dispersive spectroscopy (EDS) was performed on a JEOL 2100F microscope operating at 200 kV in scanning-transmission mode (STEM). STEM images were obtained using a high angle annular dark field (HAADF) detector (HAADF), which allows for Z-contrast imaging. Fourier-transform infrared spectroscopy (FTIR) was performed on a PerkinElmer spectrometer (Spectrum One model), using powder samples at room temperature in UATR mode (range of 4000–650 cm^–1^). Particle size, polydispersity index (PDI), and zeta potential of NaTNT and ZnTNT nanostructures in aqueous dispersions were obtained in a Zetasizer (ZEN3600, Malvern), while sodium and zinc concentrations were determined by ICP-MS (inductively coupled plasma mass spectrometry) (Agilent, 7700 model). X-ray photoelectron spectroscopy (XPS) measurements were performed by using a PHOIBOS 150 MCD-9 multichannel analyzer (SPECS GmbH, Berlin, Germany) using a detector AlKα (1486.6 eV) X-ray source. Spectra were recorded using an analyzer pass energy of 30 V, an X-ray power of 100 W, and an operating pressure of 10–9 mbar. Spectra analyses were performed using CasaXPS software with a Shirley background and symmetric Gaussian–Lorentzian line shapes. Binding energies were referenced to C 1s at 284.5 eV.

### 2.3 Preparation of the in Vitro Assays

#### 2.3.1 Experimental Design

**Figure 1:**
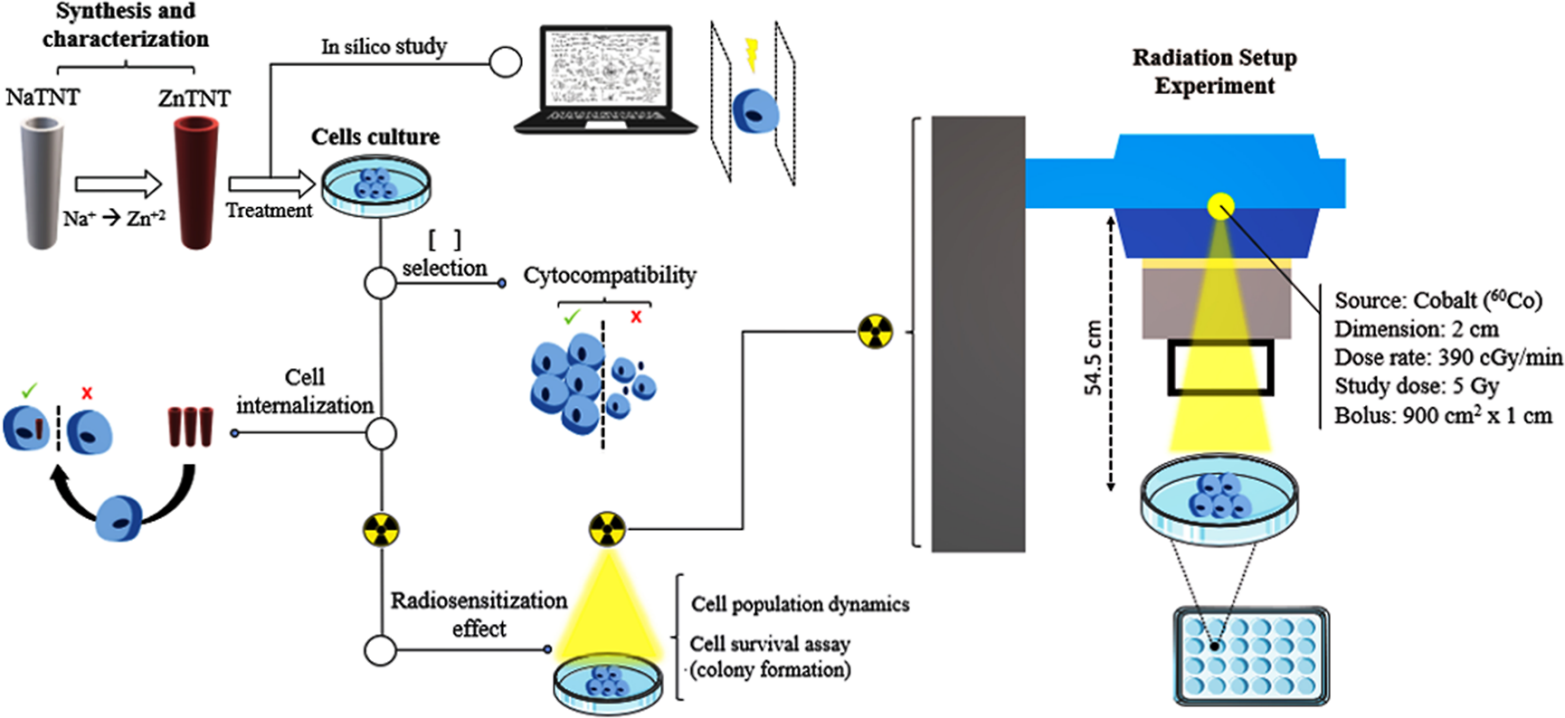
Representation of the experimental design conducted during the study.

In this study, the experiments were performed sequentially with different objectives (synthesis, characterization, and in silico and in vitro evaluations of radiosensitization activity) as described in Figure 1. Furthermore, for in vitro experimental procedures, cells were trypsinized (0.5% trypsin in 5 mM EDTA), counted on a hemocytometer, and seeded at the appropriate density, according to the experimental protocol. For each treatment condition (control, NaTNT and ZnTNT), a nonirradiated group (0 Grays, Gy) corresponded to 100% survival (control), to assess only the cytotoxic effect of ionizing radiation. All treatments conditions occurred for 24 h before irradiation. The exposure to irradiation was performed with γ radiation at the single dose of radiation (5 Gy). After irradiation, the GBM cells were incubated (37 °C, relative humidity of 95%, and 5% of CO_2_) for 24 h, before in vitro analysis.

The irradiation experiments were performed with γ radiation at a single dose of 5 Gy using a Cobalt (^60^Co) source from Theratron Phoenix (Theratronics Ltd.a., Ontario, Canada) at a distance between the source and the target of 54.5 cm.

#### 2.3.2 Preparation of ZnTNT Treatment

The preparation of TNT suspensions in pure water at the concentration (1000 μg mL^–1^) and serially diluted in DMEM in increasing concentrations (5, 15, 25, 50, and 100 μg mL^–1^). The stock suspensions were sonicated, and to ensure the uniform suspension of the treatment, they were stirred on vortex agitation before every use.

#### 2.3.3 Cell Culture Conditions

The human GBM cell lines (U87 and U251) and African green monkey kidney cell (Vero) were obtained from American Type Culture Collection (ATCC - Rockville, Maryland, USA). Cells were cultured in DMEM with 10% (v/v) FBS, 1% (v/v) penicillin/streptomycin, and 0.1% (v/v) fungizone. Cells were kept in a cell incubator (37 °C, 5% CO_2_, and 95% humidity).

#### 2.3.4 In Vitro Cell Viability

Cell viability was determined by the MTT assay.^56,57^ For evaluation of cell cytocompatibility, Vero cells were seeded at a density of 2.5 × 10^3^ cells/well in 96-well plates. After 24 h, Vero cells were treated with different concentrations (5, 15, 25, 50, and 100 μg mL^–1^) of NaTNT and ZnTNT for 72 h. Similar exposure conditions were employed to evaluate cytotoxicity in U87 and U251 cells (5, 15, and 25 μg mL^–1^). After 72 h of treatment, the medium was removed, and the cells were washed with PBS (pH = 7.2–7.4), added with 100 μL of MTT, and incubated for 3 h. The formazan crystals were dissolved in 100 μL of DMSO. The absorbance was quantified in a Spectra Max M2e (Molecular Devices) at 570 nm. The absorbance was linearly proportional to the number of living cells with active mitochondria. The results were determined as a percentage of the absorbance of the treated cells in relation to the control group.

#### 2.3.5 Evaluation of Cell Internalization

The cell internalization of ZnTNT was assessed through TEM and ICP-MS. TEM analysis was used to visualize the presence of nanostructures within the cells, while ICP-MS was used to quantify intracellular TNT levels. U87 and U251 cells were seeded in 6-well plates at 150 × 10^3^ cell/well. After 24 h, GBM cells were treated with 5 μg mL^–1^ NaTNT and ZnTNT and incubated for 24 h. To assess internalization kinetics, time intervals of 24 and 48 h were employed. After the period of exposure to TNTs, GBM cells were trypsinized, centrifuged, and washed twice with PBS (pH 7.2–7.4). Pellets were then fixed in a mixture of 4% paraformaldehyde and 2.5% glutaraldehyde buffered with 0.1 M PBS (pH 7.2–7.4) at room temperature. For TEM analysis, pellets were then postfixed in osmium tetroxide for 45 min before dehydration. The dehydration was performed in a graded acetone series (30–100%) and embedding in Araldite (Durcupan ACM, Fluka) for 72 h at 60 °C. Thin sections (100 nm) were stained with 2% uranyl acetate, followed by lead citrate. Ultrastructural analysis was performed using transmission electron microscopy (TEM, FEI Tecnai G2 T20). For ICP–MS analysis, the pellets were resuspended in pure water for the digestion process, and then the titanium (Ti) content was determined.

#### 2.3.6 Determination of the Radiosensitization Effect of ZnTNTs

The radiosensitivity of U87 and U251 cells was determined by (1) cell counting (to evaluate the biological response), (2) nuclear morphometric assay (NMA) (to determine the trend of tumor dynamic after irradiation), and (3) clonogenic assay (to evaluate the effect of radiation after 10 days), according to the methods previously described in the literature.^58,59^

##### 2.3.6.1 Evaluation of Biological Response of TNTs Combined with 5 Gy

The lineages U87 and U251 cells were seeded in 24-well plates at 10 × 10^3^ cell/well and treated with 5 μg mL^–1^ of NaTNT and ZnTNT for 24 h. The radiosensitivity of GBM cells was determined by cell counting to evaluate the proliferative response on cell numbers 24 h after irradiation. The cell number was determined in a Countess FL cell counter (Life Technologies) using the trypan blue dye exclusion protocol.^60^ The results were expressed as percentage of live cells in relation to the nonirradiated control group.

##### 2.3.6.2 Evaluation of Cells Dynamic after Irradiation

The tumor cell dynamics were determined using NMA, as described by Filippi-Chiela et al.^61^ NMA is a straightforward approach that assesses cell fate (apoptosis, senescence, or mitotic catastrophe) based on nuclear alterations, including shape and size. U87 and U251 cells were seeded in 24-well plates at 10 × 10^3^ cell/well and treated with 5 μg mL^–1^ of NaTNT and ZnTNT for 24 h. The NMA protocol was performed 24 h after irradiation, and the nuclei were fixed with 4% paraformaldehyde and stained with Hoechst at a dilution of 1:1000 in PBS. Images were acquired in a fluorescence microscope, followed by analysis in the Image-Pro Plus 6.0 software (IPP6, Media Cybernetics) for the acquisition of nuclear variables (area, radiusratio—Rr, roundness—Rou, aspect—Asp, and areabox—Arbx). The nuclear shape is defined by the nuclear irregularity index (NII), which is calculated by the following formula: NII = Asp – Arbx + Rr + Rou as described by Vargas et al.^62^ The results were presented as a plot of area versus NII. The NMA classifies the nuclear as normal (N), small and regular (SR), small and irregular (SI), large and regular (LR), and large and irregular (LI). Typically, SR nuclei correspond to apoptotic cells, while LR and LI are indicative of nuclei from senescent cells.

##### 2.3.6.3 ZnTNT Induced Radiosensitivity

The radiosensitivity simulating a clinical response (survival fraction) was determined by clonogenic assay, as previously described.^63^ The radiosensitivity of U87 and U251 was determined after treatment with 5 μg mL^–1^ of TNTs and irradiation (5 Gy). The irradiated GBM cells were recultured in 6 well plates (2 × 10^2^ cells/well) and maintained in culture for 10 days. At the end of the experiment, the cells were washed with PBS, fixed with 4% formaldehyde for 20 min, and stained with methylene blue for 10 min. At the sequence, cells were washed two times with PBS and dried at room temperature. The results were demonstrated as absolute number of colonies.^59,60^

### 2.4 Monte Carlo Computational Simulations

**Figure 2:**
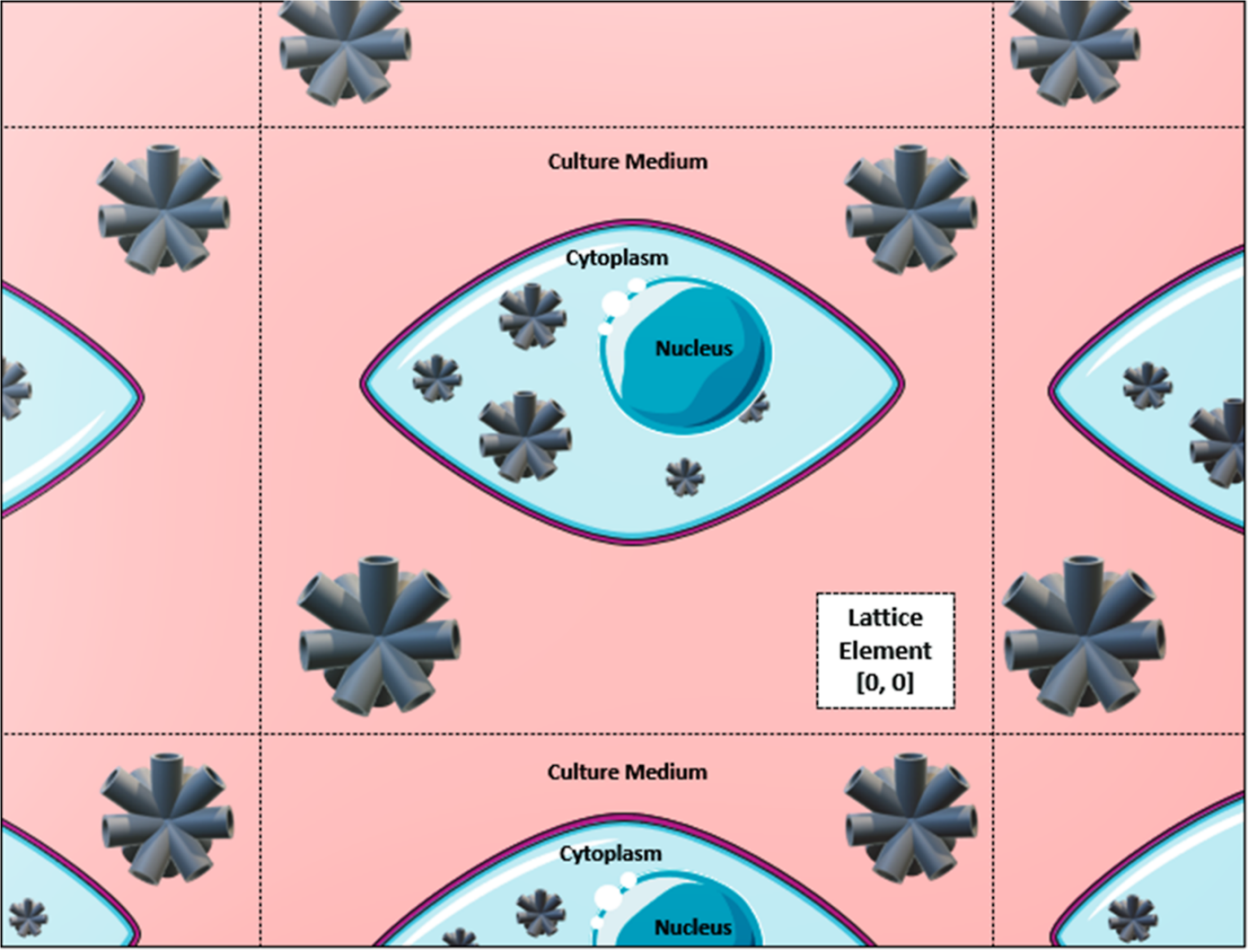
Schematic of the cell lattice used for the simulations. Region colored by red represents the cell culture medium, in light blue is the cell cytoplasm, in dark blue is the nucleus, and in gray are the TNT clusters.

The MCNP code version 6.2 by LANL and the Evaluated Nuclear Data File (ENDF/B–IV.8) cross-sections were used to calculate the interactions of photons and electrons with the matter that compose the cells, the tubes, and the cell culture medium.^64^ The ^60^Co gamma source emits photons of two energies, with an average energy of 1.250 keV. The cut-off limits were set at 250 eV electron transport and 1 keV for source photons; in addition, the physics of electron transport was also modified. The production of Bremsstrahlung photons, especially photon-induced secondary electrons such as photoelectrons and Auger and knock-on electrons, was adjusted. Moreover, the control stopping power energy spacing was lowered to obtain a better special resolution for tracking secondary electrons. The cell culture medium was approximated to an aqueous medium; for simulation purposes, it does not take large computation time. Each well used as culture medium was represented by a cylinder with 1.625 cm of diameter, and the lower and upper layers of this well were composed of polystyrene with density of 1.06 g/cm^3^. A lattice with 256 (16 × 16) elements, where each of these elements corresponds to a set of cell and TNT clusters, was built to improve the statistics of computer simulation results. Although this work presents two distinct types of cells, computer simulations were constructed by using mean values of cell sizes and TNT internalization concentrations. The cells had an elliptical shape, with a larger diameter of 100 μm and a smaller diameter of 60 μm; the spherical cell nucleus had a diameter of 30 μm;^65,66^ and the TNT clusters had also a spherical shape, with sizes between 5 and 40 μm.^23^ The distribution of TNT clusters in the intracellular and extracellular media was random, taking into consideration the internalization values. Figure 2 shows a representation of the cell scenario.

For cell composition, the International Commission on Radiological Protection (ICRP) soft tissue was used as reference, and its density was 1.04 g/cm^3^.^64,67,68^ The culture medium and the compensating bolus were defined as the equivalent water with a density of 0.99 g/cm^3^. Each cell present in the matrix was tallied by energy deposition primarily from photoelectrons and Auger electrons with energies between 8.5 eV and 225.5 keV and in the range between 0.25 nm and 210 μm. Some tallies, such as + F6 and *F8, were used to register the energy deposited values in units of megaelectron volts per starting particle. In order to physically evaluate the increase in the radioenhancement due to nanotubes, the dose enhancement factor – DEF – was used. DEF (eq 1) is defined as the factor by which the deposited dose is increased due to the presence of nanotubes.^69,70^
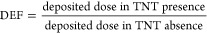
1

### 2.5 Statistical Analysis

The in vitro experiments were repeated at least four times for each concentration, all in triplicate. Results were expressed as standard error of the mean and analyzed for statistical significance by one-way analysis of variance (ANOVA) followed by Tukey posthoc test (Prims GraphPAD 8.0). Values of *p* < 0.05 were considered statistically significant.

## 3 Results and Discussion

Tumor therapy by ionizing radiation requires attention, since it is related to the possibility of not completely eradicating the tumor, limiting the success of treatment.^7,16,71^ For this reason, the interest in nanostructures such as TNTs has grown notably in recent years due to their biocompatibility and ability to radiosensitize tumor cells. Studies combining TNT with RT are still scarce in the literature. In this study, aiming to contribute with nanomedical studies for GBM treatment, we synthesized ZnTNT to improve the radiosensitization effect in GBM cells.

### 3.1 ZnTNT Characterization

**Figure 3:**
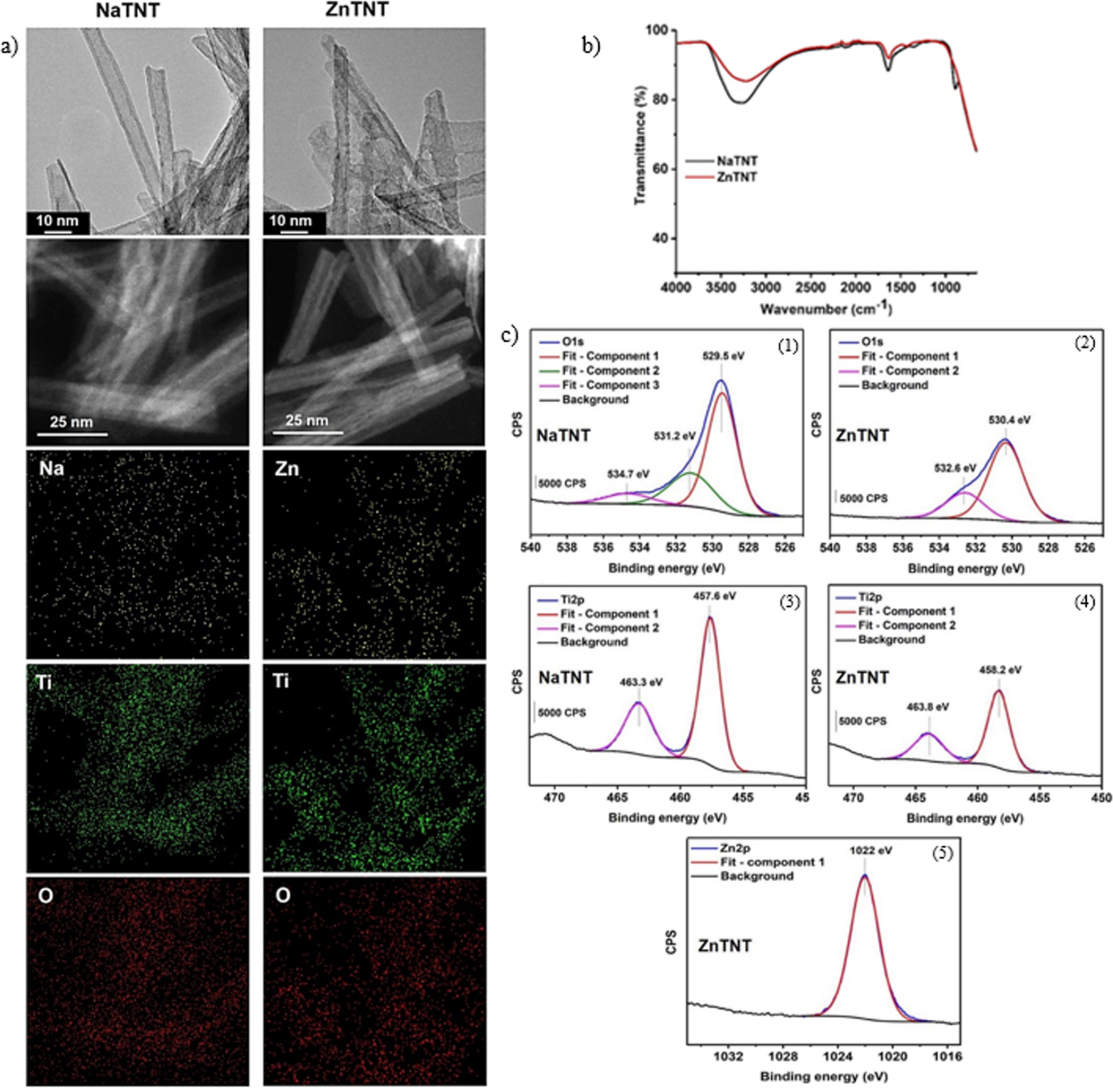
Characterization of NaTNT and ZnTNT. (a) TEM, SEM, and EDS results of NaTNT and ZnTNT. (b) FTIR spectra of NaTNT and ZnTNT nanostructures. (c) XPS spectra with emphasis in the oxidation state (1, 2), Ti–O bond (3, 4), and Zn–O bond (5).

TEM and STEM results (Figure 3a) showed that sodium TNTs were formed by folding at least three nanosheets, resulting in a tubular structure and an external diameter of 9.0 ± 0.5 nm. After ion exchange, nanotubes modified with Zn have an irregular surface and external diameter to 8.1 ± 1.8 nm. Elemental mapping (Figure 3a) indicated an uniform distribution for the main elements, i.e., Ti, O, and Na, that constitute the basic nanostructure of the NaTNT. For ZnTNT nanostructures, the mapping showed the Zn element presence uniformly over the whole surface of the modified TNT, indicating a good distribution of this element and confirming ion exchange.^72^

Another way to evaluate ion exchange (from Na to Zn) is analyzing these nanostructures by FTIR. NaTNT presented three characteristics bands (Figure 3b), located between 3500 and 3200 cm^–1^ attributed to hydroxyl groups (O–H) adsorbed on the TNT surfaces, 1640–1630 cm^–1^, corresponding to the O–H deformation,^73,74^ and a band located at 900 cm^–1^ assigned to the vibrational mode of the Ti–O bond (no bridging oxygen atoms coordinated with Na^+^ ions).^75,76^ ZnTNT nanostructure showed a similar FTIR spectrum to NaTNT, except by the band disappearance in 900 cm^–1^, corroborating the ion exchange of Na by Zn already observed in EDS mapping.

These results were also corroborated by ICP analysis, where it was determined that the Na concentration was 9.04% in the NaTNT. After the exchange of Na by Zn, this value decreased to 0.12%, whereas the concentration of Zn was 6.8%, confirming that there was an effective ion exchange. A relatively smaller amount of Zn was incorporated in the tubular titanate nanostructure, probably because this element was located essentially on the surface of nanotubes.

Hence, to ascertain the efficacy of ion exchange (Na^+^ → Zn^2+^) and the ensuing nanoparticle formation, we meticulously scrutinized the mean dimensions, PDI, and surface charge, with the findings tabulated in Table 1. After the ion exchange process, a discernible expansion in nanoparticle hydrodynamic diameter was noted (a parameter for understanding the behavior of nanoparticles in solution, especially in biological research). The PDI values demonstrated a modest escalation following the introduction of Zn (ranging from 0.27 to 0.33), reflecting a relatively consistent particle distribution across the analyzed specimens. Finally, NaTNTs presented a surface charge of −35.6 ± 0.28 mV, while ZnTNTs show a charge of +16.8 ± 0.26 mV, measured by zeta potential. Negative charge observed on NaTNT occurs due to the presence of a partially hydroxylated surface. A similar observation was made by other authors.^77,78^ After modification with Zn, an inversion in the zeta potential was observed. This result indicates an effective substitution from Na^+^ to Zn^2+^ ions, reinforcing previous observations, as well as indicating the Zn–O interaction on the nanotube surface by this atom. As described in this study, an inversion in zeta potential was also observed after functionalization of TNTs with cationic groups in the literature.^79,80^

**Table 1:** Summary of Size, PDI, and Zeta Potential Values for NaTNT and ZnTNT

sample	size (nm)	PDI	zeta potential (mV)
NaTNT	269.6 ± 9.2	0.31 ± 0.05	–35.6 ± 0.4
ZnTNT	248.6 ± 10.4	0.47 ± 0.03	+16.8 ± 0.4

XPS analysis (Figure 3c) was carried out in order to evaluate the chemical state of elements (i.e., Ti and Zn) and lattice oxygen presented in TNT nanostructures. The signal of O 1s presents three different components with values of bidding energy at 534.7, 531.2, and 529.5 eV, which can be assigned to H_2_O, surface-bound hydroxyl groups (in Ti–OH) and lattice oxygen Ti–O (from Ti–O–Ti), respectively.^81,82^ The signal corresponding to H_2_O is not observed for ZnTNT and shifts of 0.9 eV observed for the other signals are due to O binding that presented new interaction,^83,84^ possibly with Zn corroborating the result obtained from the zeta potential.

The Ti 2p XPS spectra of NaTNT and ZnTNT showed two peaks around 463 and 458 eV that are characteristic of 2p_3/2_ and 2p_1/2_ spin doublet from Ti^4+^.^85^ The ZnTNT nanostructure presented a signal located at ∼1022 eV indicating the Zn^2+^ oxidation state and a chemical composition of pure metallic oxide.^86^

### 3.2 In Vitro Biocompatibility and Cytotoxicity of ZnTNTs

**Figure 4:**
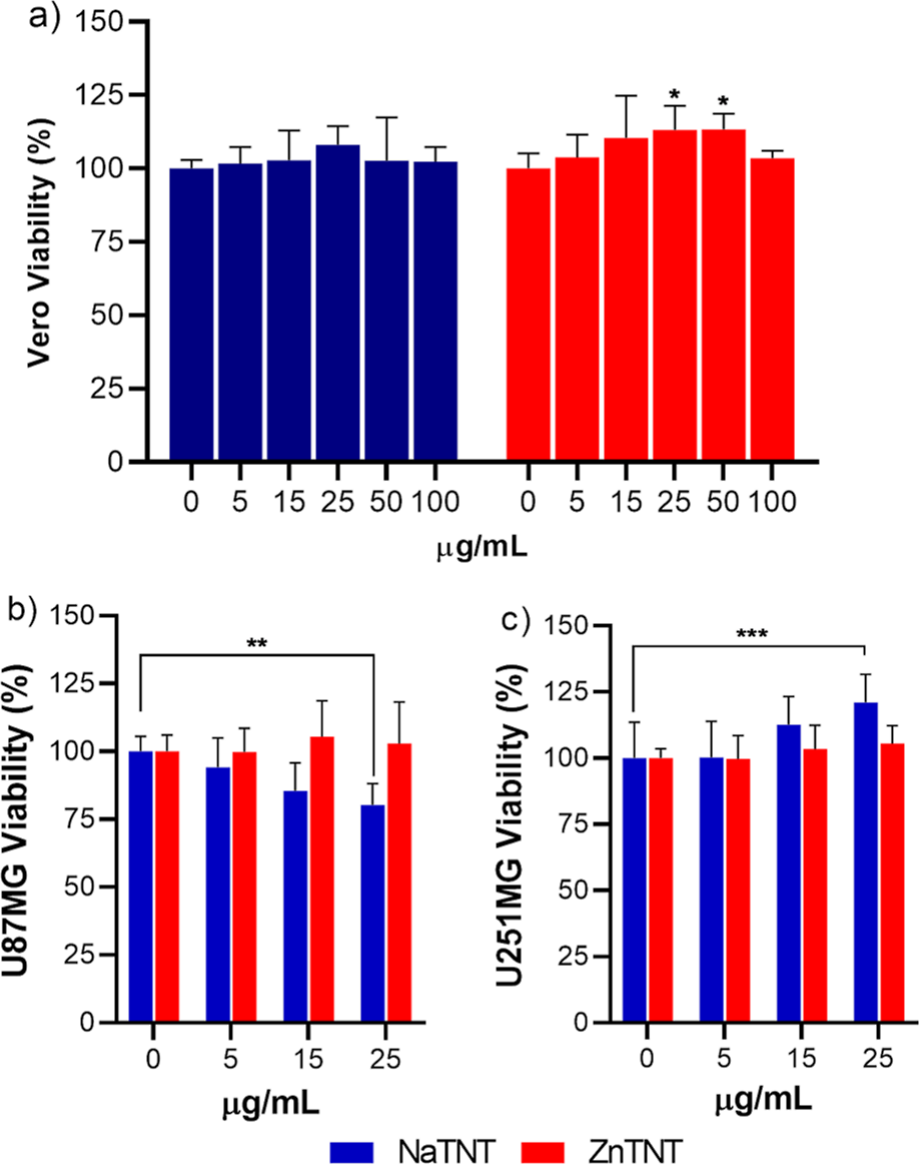
Effect of NaTNT and ZnTNT on viability of Vero, U87, and U251 cells. After confluence, (a) Vero, (b) U87, and (c) U251 cells were treated with different concentrations of TNTs for 72 h. The data were analyzed for statistical significance by one-way ANOVA, followed by Tukey posthoc. ***p < 0.001, **p < 0.01, *p < 0.05.

First, a biocompatibility study was conducted to determine maximum tolerance of healthy cells to zinc-titanate and sodium-TNTs. Nanotube concentrations were extrapolated to measure a safe margin noncytotoxic in Vero cells. Cells were exposed to a range of concentrations from 5 to 100 μg mL^–1^ of NaTNT or ZnTNT for 72 h (Figure 4a).

As shown in Figure 4a, none of the nanostructures were able to alter the viability of Vero cells at tested concentrations. MTT results indicated that NaTNT and ZnTNT were considered noncytotoxic after 72 h. This result is consistent with previous studies that demonstrated a biocompatible profile of TNT for healthy cells.^23,80^ Sruthi et al.^80^ conducted studies with TNT on microglial cells and reported a nontoxic profile attested concentrations after 24 h. Besides, Alban et al.^23^ reported biocompatibility of NaTNT in Vero cells after 48 h. In our study, preservation of the atoxic profile after ion exchange was observed. Biocompatibility profile of both TNTs was evidenced in vitro model study with Vero cells after 72 h. This group of cells is used for toxicity evaluation of chemical compounds at a molecular level.^87^

Once a nontoxic profile of NaTNT and ZnTNT was observed in healthy cells, the effect of nanostructures on GBM cells (U251 and U87) was evaluated (Figures 4b,c). This experiment was conducted with two proposals: to determine the concentration for the next experiments and assess the cytotoxic effect of nanostructures in the absence of irradiation.

MTT results showed reduced viability (20%) in U87 cells treated with NaTNT (25 μg mL^–1^) at 72 h (Figure 4b). A greater mitochondrial metabolism can explain last results; however, more studies are necessary to understand this point. Cell viability increase was observed in U251 treated with NaTNT (25 μg mL^–1^); however, in other treatment conditions, no viability change was found in response to both TNTs (Figure 4c).

These findings from cytotoxicity activity were similar to those found in other studies.^18−20,88^ Results indicate that TNTs were not capable of reducing tumor viability, except when loaded with drugs.^19^ Regarding the mentioned proposition, our starting point for this research was to comprehend the interaction between nanostructures with cells (normal and tumor) using an in vitro study model to establish a concentration capable of interacting with biological material without toxicity damage. The noncytotoxic dose of 5 μg mL^–1^ of TNTs was chosen for both cell lines to perform subsequent experiments.

### 3.3 Kinetics of Cellular Uptake of ZnTNTs

**Figure 5:**
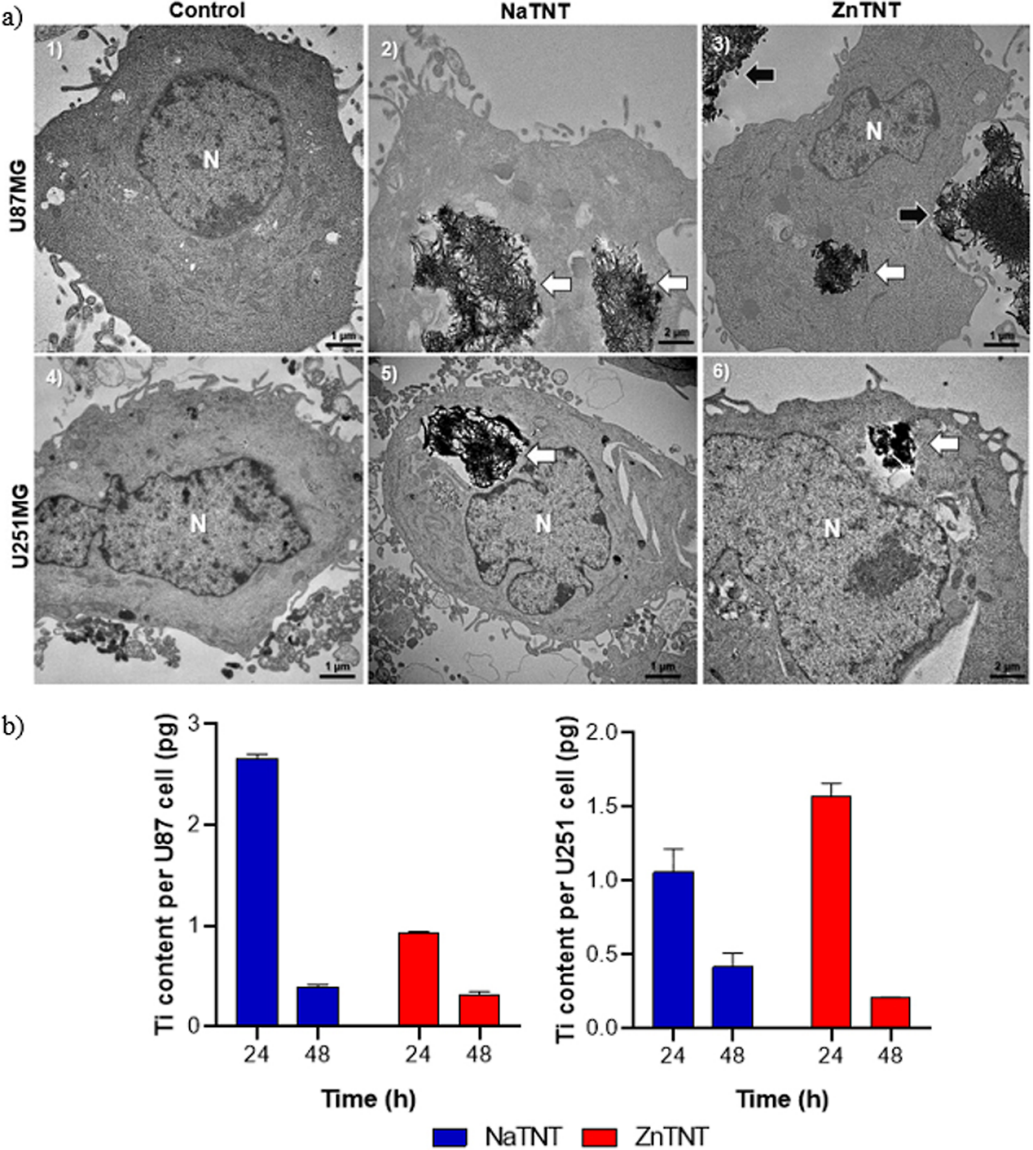
TNT cell internalization study. (a) TEM images of GBM cells incubated for 48 h with TNTs (5 μg mL^–1^). (1) control U87 cell; (2) high rate of NaTNT internalization inside the U87 cell (white arrow); (3) ZnTNT nanostructures were detected into the cytosol of U87 (white arrow) and outside the cells after washing (black arrow); (4) control U251 cell; (5) high rate of NaTNT detected into the cytosol (white arrow); and (6) high rate of ZnTNT inside vesicles into U251. (N—nucleus). Study of internalization was performed by ICP-MS. (b) Ti content in pg cell^–1^ in U87, and U251 after 24 and 48 h of exposure.

Internalization of nanomaterials is an important event in terms of bionanointeraction,^20,80^ and it is known that several characteristics are involved on cellular internalization of materials, such as diameter, length, shape, volume, surface charge, and functionalization.^89−92^ Thus, after determining the concentration of the experiment, we also evaluated NaTNT and ZnTNT internalization in U87 and U251 cells by TEM (Figure 5a) and ICP-MS (Figure 5b,c).

Images obtained by TEM show a high rate of TNTs internalized by GBM cells after 48 h of the incubation and washing process (from sample preparation to TEM). Some studies reported internalization of TNT in GBM,^18,20^ bladder cancer,^23^ prostate cancer,^19^ cardiomyocytes,^78^ and microglial cells.^80^ In this study, the nanostructures were observed inside the vesicle (white arrow; Figure 5a.2,a.5,a.6) and in cytosol (white arrow; Figure 5a.3), inside GBM cells. The first process described may occur by a passive and spontaneous process of diffusion through the plasma membrane, while the second one occurs via endocytosis as an active process.^89^ Moreover, the presence of NaTNT and ZnTNT outside U87 and U251, despite washing steps, suggests the possible exocytosis of TNTs (black arrow; Figure 5a.3).

In general, nanosized systems are better internalized by cells than by larger particles. Furthermore, higher cellular uptake occurs in tubular nanomaterials when compared with spherical ones.^77,93^ Both NaTNT and ZnTNT exhibited tubular morphology formed by winding at least three titanate multilayer lamellar walls, as described previously.^18,23^ Thus, NaTNT and ZnTNT can realize a direct membrane penetration as an individually dispersed nanotube, behaving like a nanoneedle (Figure 5a.3). This process could be responsible for a minimal amount of cellular internalization of the TNTs. As can be observed (Figure 5a.2,a.5,a.6), cellular internalization via endocytosis represents the most important pathway in terms of volume of TNTs.^18,19,23^ This process first occurs by the adhesive interaction between nanotubes and cell membrane mediated by hydrogen bonds, van der Waals, and electrostatic forces.^77,90,94,95^

The ICP-MS was used to quantify the intracellular concentration of Ti after 24 and 48 h of GBM cells incubation with both nanotubes. As shown in the Figure 5b,c, the intracellular Ti content increased in the first 24 h of incubation and decreased in 48 h in both GBM cells. NaTNT concentration increased in U87 cells, while ZnTNT was higher in U251 cells after 24 h. After 24 h of incubation, the results indicate that there is a greater accumulation of TNTs in the intracellular region. Nevertheless, additional research is required to elucidate whether there is a saturation point in the internalization of TNTs, along with the presence of a feedback mechanism that encourages the continuous uptake of nanostructures.

**Figure 6:**
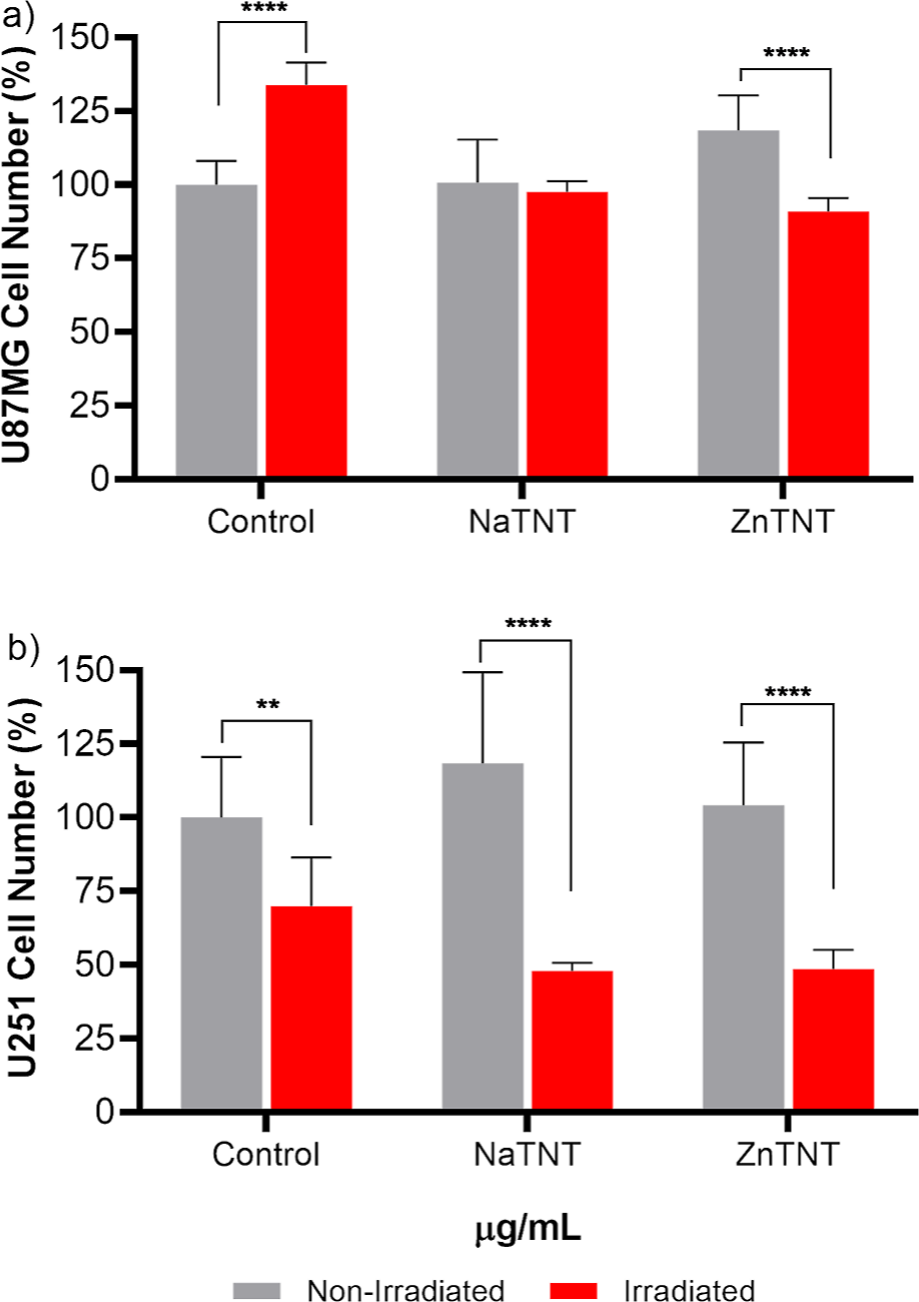
Effect of TNTs on the proliferation of the U87 and U251 cells line. At 80–90% of confluence, (a) U87 and (b) U251 cells were treated with 5 μg mL^–1^ of respective nanostructures for 24 h. Next, one group was irradiated (5 Gy) and another group was not (0 Gy). 24 h after irradiation, cells were detached and counted. Non-irradiated control were considered as 100%. Data were analyzed for statistical significance by one-way ANOVA, followed by Tukey’s posthoc. ***p < 0.001.

**Figure 7:**
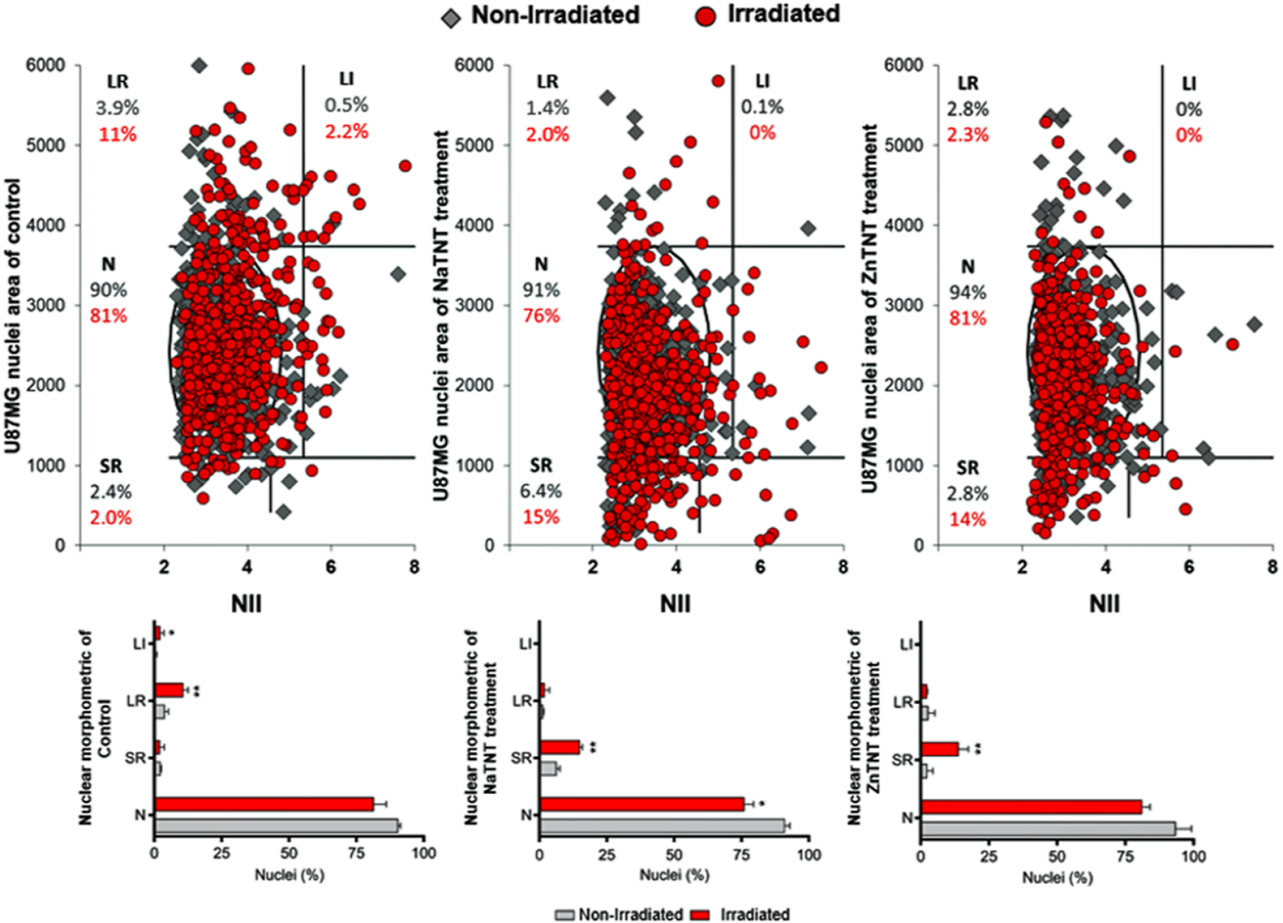
NMA graph of nonirradiated and irradiated U87 cell line after 24 h of irradiation. The values on the bars graph represent percentage of cells for respective treatment. The data were analyzed for statistical significance by one-way ANOVA, followed by Tukey posthoc. ***p < 0.001, **p < 0.01, *p < 0.05.

**Figure 8:**
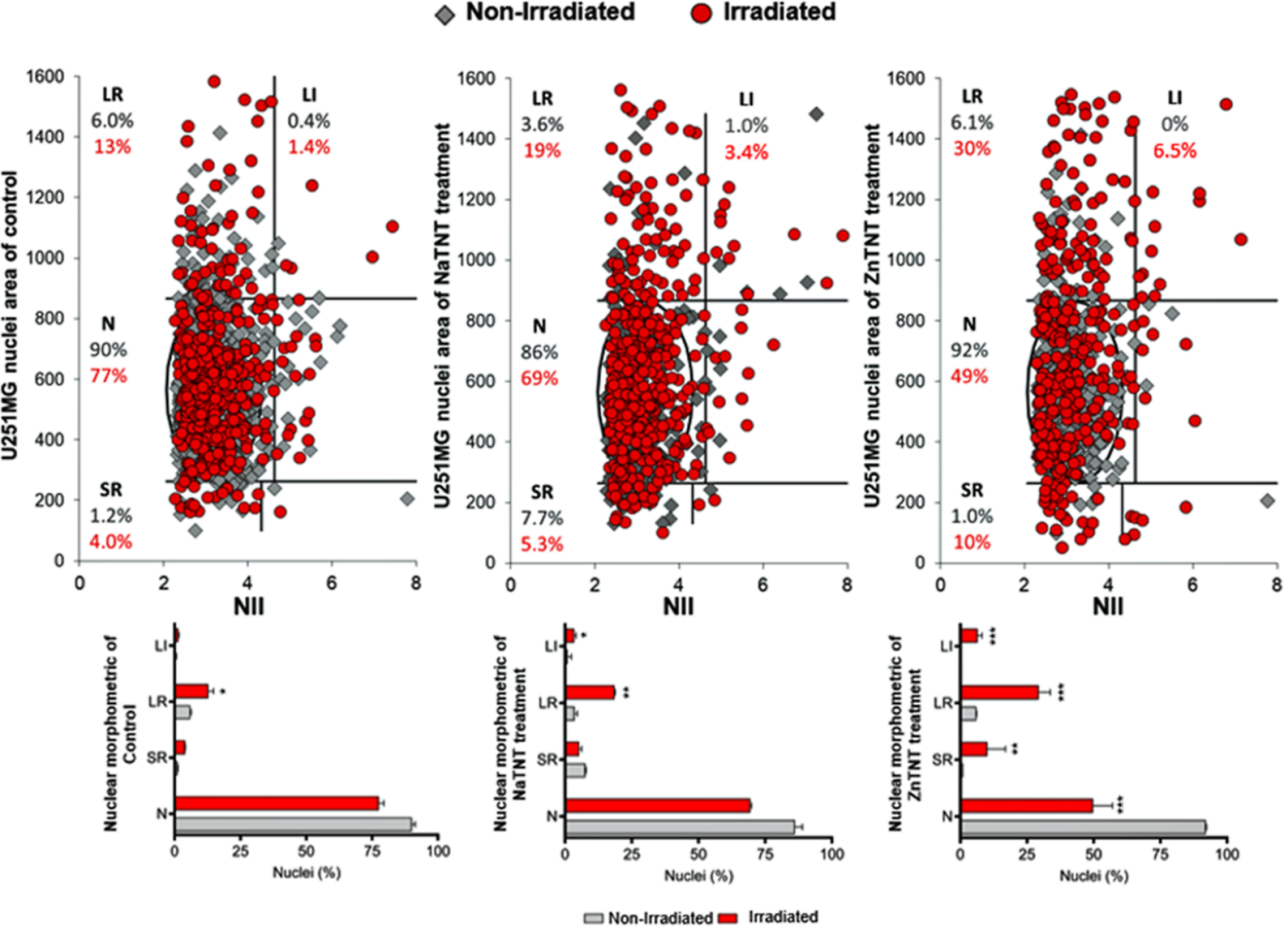
NMA graph of nonirradiated and irradiated U251 cell line after 24 h of irradiation. The values on the bars graph represent percentage of cells for respective treatment. The data were analyzed for statistical significance by one-way ANOVA, followed by Tukey posthoc. ***p < 0.001, **p < 0.01, *p < 0.05.

Nanotubes with a positive surface charge are likely to interact with the slightly negatively charged cell membrane. This interaction can lead to increased flexibility in the lipid bilayer of the membrane, which in turn facilitates their uptake through a process called adsorptive endocytosis. This ultimately results in the formation of bundles of nanotubes within vesicular compartments.^90,93,96−98^ This observation could justify a possible greater internalization of ZnTNT when compared to NaTNT in U251 cells since ZnTNT has a positive charge demonstrated by zeta potential, but further studies are needed. In order to reinforce our findings, several authors have demonstrated both pathways of internalization from TNT in different types of cell lines.^19,23,78,80,92^ Furthermore, the ultrastructures of U87 and U251 cells were observed by TEM (Figure 8). These TEM results suggest that treatments with NaTNT and ZnTNT did not promote changes in the nuclear aspect, compared to the nuclei of cells not treated with TNTs, indicating the absence of toxic effects.

### 3.4 Effects of ZnTNT Combined with Irradiation

RT can act by inhibiting and controlling growth, proliferation, and metastasis in malignant cancer cells through the deposit of high-energy radiation on tumor tissues.^71,99−101^ Ionizing radiation delivered in RT can trigger a cascade of physical, chemical, biological, and clinical events and even lead to cell death from irreparable DNA damage.^102−105^ To evaluate the possible potential of nanostructures in reducing the viability of tumor cells in combination with RT, a cell counting assay was carried out (Figure 6). Furthermore, NMA was used to understand and determine the behavioral and morphological profile of therapy-surviving tumor cells (Figures 7 and 8) after 24 h of exposure to irradiation. Thus, GBM cells lines were incubated with NaTNT and ZnTNT for 48 h. After this period, one group from each cell line was exposed to a γ radiation dose of 5 Gy, while another group was not exposed (0 Gy).

The radiation effect was observed by reduction of living cells 24 h postirradiation (5 Gy). Although TNTs are not cytotoxic, results showed that NaTNT and ZnTNT when combined with a RT dose (5 Gy) were able to reduce proliferation in U87 (around 20 and 25%, NaTNT and ZnTNT, respectively) and U251 (53 and 52.5%) (Figure 6), corroborating with previous findings.^23^ On the other hand, in the absence of radiation, our results demonstrated that neither type of nanotubes was able to significantly change proliferation rates in GBM cells (Figure 6), which suggest the value of combined therapy to enhance the effect of RT. The difference in isolated irradiation response between GBM lines could be explained to the fact that U251 is less radioresistant than U87.^106^

Previously, a study has demonstrated radiosensitization effect of TNTs in bladder cancer cells.^23^ Alban et al. reported that NaTNT and ZnTNT reduced the number of live cells in T24 tumor cells when combined to ionizing radiation, with NaTNT nanostructures being more effective in inhibiting tumor proliferation than ZnTNT.^23^ In our study, both nanostructures induced similar biological response in different GBM cells, which was also previously observed in human bladder tumor cells; however, we used a concentration of TNTs five times lower than used here.

#### 3.4.1 Effects of Tumor Dynamics after the Combination of ZnTNT and Irradiation

NMA is a tool that is able to analyze the alteration in nuclear morphology that occurs in several cellular processes, like during senescence (increase in nuclear size) and apoptosis (nuclear condensation and fragmentation).^61^ NMA was performed for a better understanding behavioral/morphological profile of therapy-surviving tumor cells 24 h postirradiation (Figures 7 and 8).

As presented in Figure 7, nonirradiated U87 cells did not show any significant nuclear alteration in the response profile among the control group, NaTNT, and ZnTNT. However, the same cells exhibited an increase of SR nuclei (SR) around 16 and 14% when 5 Gy irradiation dose was combined to NaTNT and ZnTNT, respectively. Nucleus alterations observed in NMA analysis may be related to the morphological characteristics of apoptotic cells (Figure 7, irradiated group). Apoptosis is an organized process, which leads to cell death; this phenomenon is characterized by the high and regular condensation of the nucleus.^107−109^ Furthermore, irradiated U87 cells showed a significant increase in nuclear enlargement (suggestive of senescent phenotype).^60,61^ On the other hand, the number of cells with normal characteristics remained practically unchanged. Only the irradiated group combined with NaTNT showed a significant reduction in the percentage of normal cells.

Cells with senescent phenotype characteristics were noted in U251 cells subjected to ionizing energy. In the NMA analysis, nonirradiated U251 cells showed no change in nuclei morphology, as expected in this study (Figure 8). Moreover, in the irradiated group, an increase of large and regular nucleus (LR) percentual was possible to observe in all experimental conditions of the irradiated group. However, samples containing TNTs showed higher percentages of cells with LR characteristics (13.5% for NaTNT; and 29% for ZnTNT).

The senescence induction is regarded in cancer cells as a means to halt tumor initiation and progression (because of irreversible growth arrest),^110^ and cells undergoing senescent suffer a high and regular enlargement of the nucleus.^61^ Liu et al. showed this effect in cells irradiated combined to gold nanoparticles, in which surviving cells presented postirradiation senescence morphology, namely, cell size increased significantly.^111^

Furthermore, in this work, the ZnTNT nanotubes showed better biological action than NaTNT nanotubes when combined to irradiation on U251 cells. ZnTNT combined with 5 Gy showed an increase in percentage of cells with SR and LR characteristics when compared to the irradiated control group, while the NaTNT-irradiated group showed a great reduction in number of normal nuclei. One possible explanation would be related to the internalization rate of ZnTNT due to the fact that a high amount of internalized nanostructures would increase the RT dose absorption. Another point that can help in this higher internalization is given positive charge of ZnTNT, what does not happen with NaTNT.^112,113^ However, further studies need to be carried out to better understand this result.

The greatest contribution of this analysis is to demonstrate the behavioral tendency of a group of tumor cells under the same conditions and how they react differently to a specific treatment. This assay also makes it possible to characterize the tumor complexity and the dynamic profile of individual tumor cells^114,115^ and allows for a better understanding of the disease recurrence, as well as identifies where the fails of therapy are.

#### 3.4.2 ZnTNT Induced Radiosensitivity

**Figure 9:**
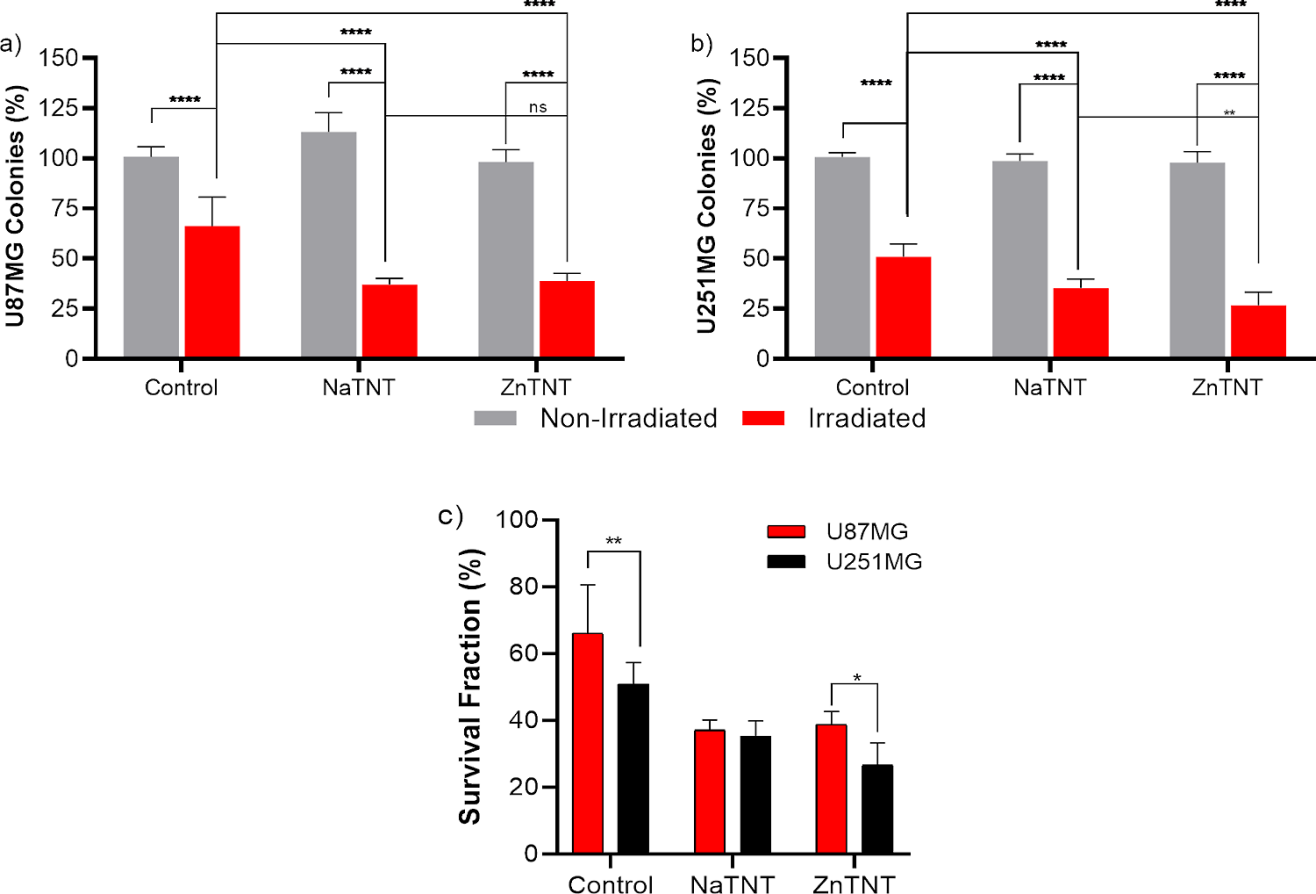
Ability of U87 and U251 cells to form new colonies after 5 μg mL^–1^ TNT treatment with 5 Gy. A clonogenic assay was performed to assess TNT combined with irradiation on cell proliferation after 10 days. Quantification of percentage of U87 (a) and U251 (b) colonies. Comparison of fraction of survival of U87 with U252MG (c). Each column represents the mean ± SEM, ***p < 0.001, **p < 0.01, and *p < 0.05, and the results were established in relation to control cells. The experiments were performed in triplicate.

The concept of cell death is related to the irreversible cessation of vital functions (loss of clonogenic integrity and unable to proliferate indefinitely).^116,117^ The potential effect of TNT-induced radiosensitivity is related to the ability of TNT and irradiation combination to affect clonogenic proliferation. The radiosensitization effect of NaTNT and ZnTNT (5 μg/mL) combined to 5 Gy on GBM cells was assessed by clonogenic assay after 48 h of TNT cell incubation (Figure 9). In this work, only the cytotoxic effect of ionizing radiation was evaluated comparing nonirradiated with irradiated group, accepting controls of nonirradiated group, which correspond to 100% of survival.

The results indicated a significant decrease in the clonogenic integrity for GBM cell lines exposed to NaTNT and ZnTNT combined with irradiation (Figure 9a,b). When we observed the results 10 days after treatment with TNTs following irradiation, there was a significant decline in the number of polyclonal colonies of the U87 and U251 cells when compared to their respective controls (Figure 9a,b).

In the U87 cell line, we observed a statistically significant difference (*p* ≪ 0.0001) between the irradiated group exposed to NaTNT and ZnTNT when compared to the irradiated control group. On the other hand, no statistically significant differences were found between the NaTNT and ZnTNT within the irradiated group (*p* > 0.05). Conversely, in the U251 cell line, we identified a statistically significant difference (*p* ≪ 0.0001) between the treated and nontreated irradiated groups. Notably, there was also a statistically significant difference (*p* < 0.008) observed between the NaTNT and ZnTNT groups within the irradiated group. These variations in irradiation responses between the U87 and U251 cell lines suggest that the effectiveness of NaTNT and ZnTNT treatments, when used in conjunction with radiation therapy, may be contingent upon the specific tumor context. These findings underscore the significance of tailored treatment approaches in the realm of cancer therapy and emphasize the necessity for further investigations to elucidate the underlying mechanisms responsible for these discrepancies.

The survival fraction was also evaluated for each treatment condition involving TNTs, in comparison to the control group subjected to irradiation, by measuring the suppression of colony formation (Figure 9). As shown in Figure 9c, NaTNT and ZnTNT induced a significant radiosensitization effect in both cell lines. After applying these treatment conditions, the percentage of U87 survival fraction ranged from 66.2% (control) to 37.1% (NaTNT) and 38.8% (ZnTNT), whereas the percentage of U251 survival fraction ranged from 50.9% (control) to 35.3% (NaTNT) and 26.7% (ZnTNT).

As shown in the results of radiosensitization, both TNTs promoted a reduction in the surviving fraction for the GBM cells. However, the difference between two experimental radiosensitivity results were observed for U87 and U251 (Figure 9c). For U87 cells, despite the significant reduction in proliferation capacity promoted by NaTNT (decrease of 62.9%), there was no significant difference when compared with effects of ZnTNT (61.9%) in the same cell line (Figure 9a). On the other hand, in U251 cells, both TNTs promoted a reduction in proliferative integrity, but ZnTNT (decrease of 73.3%) was able to exert a significant inhibitory effect on the capacity of cell proliferation compared to that promoted by NaTNT (64.7%) (Figure 9b).

These results are similar to literature,^17−19,23^ which reported that TNTs with sodium are capable of inducing cell cycle stop of GBMs through the production of ROS,^18^ as well as tumor radiosensitization by another nanostructures.^1,16,111,118^ While a limited number of studies have explored modified TNTs for biomedical purposes, one such study conducted by Alban et al. specifically highlighted the radiosensitization effect on human bladder tumor cells.^23^

Furthermore, the present study shows the synergy between TNTs and zinc in antiproliferative capacity when combined with X-ray. The radiosensitizing effect of TNTs in the anatase form is reported in some studies, due to the surface photocatalytic effect induced by X-rays.^36^ Also, the toxicity of zinc-based nanostructures in tumor cells is associated with increased concentration, morphology, and size.^119−121^ Another possible mechanism may be correlated with the surface charge (Ann et al., 2015).

The surface charges of the zinc TNTs were examined by measuring the zeta potential (Table 1). Potentials of low magnitude (±30 mV) indicate more unstable particles, and negative signs on the surfaces suggest a tendency for positive charge flow on the surfaces of zinc-based nanostructures, while potentials with magnitudes greater than +30 mV and −30 mV indicate high stability, where the presence of the sodium cation confers stability to the nanotube structure.^121^ ZnTNT exhibited lower zeta potential and, consequently, less stable nanostructures in suspension form, probably associated with a high level of zinc ion release (Zn + 2). The data obtained by zeta potential suggest that ZnTNT acts as a ROS-generating structure and transports zinc ions, leading to a Zn + 2 delivery system for cells, which induces an increase in oxidative stress generated by ROS and results in reduced proliferation.^122^

### 3.5 MCNP6.2 Simulations

**Figure 10:**
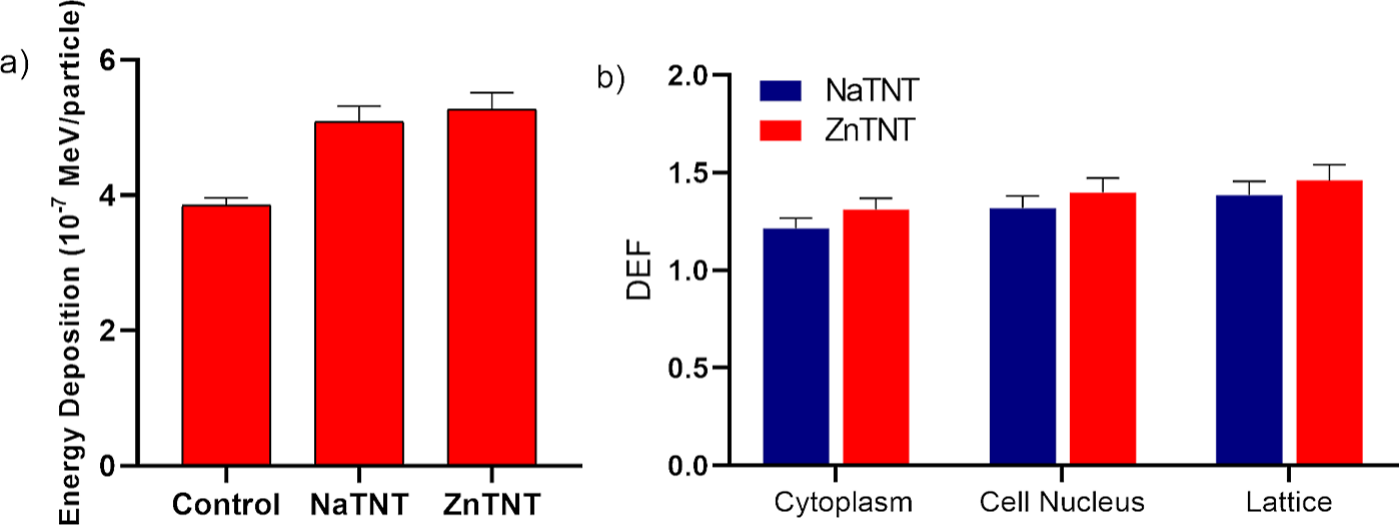
Average values of energy deposition in the control and NaTNT and ZnTNT groups (a). Average of DEF values for cytoplasm, nucleus, and whole inner cells of the lattice (b).

Metallic elements such as titanium have an increased probability of photoelectric absorption compared to light elements that compose human tissues, which leads to a greater production of low energy electrons (photoelectrons and Auger electrons). All these energy transfer processes are responsible for increasing the energy deposition around the nanotubes, producing free radicals and causing direct and indirect damage to cell DNA.^123^Figure 10a shows average dose deposition of 196 inner elements from a 16 × 16 lattice; 2 rows of outer elements were disregarded to avoid edge effects between culture medium and cells. For the control group, the deposited dose was (3.852 ± 0.116) 10^–7^ MeV/particle, while for the culture medium with ZnTNT, it was (5.273 ± 0.245) 10^–7^ MeV/particle and (5.087 ± 0.231) 10^–7^ MeV/particle for NaTNT; the percentage difference between the control and ZnTNT groups was 27%, while between the control and NaTNT groups, it was about 24%. Even though the values are relative to the number of particles from the radiation source, these results show the expected behavior trend: the presence of nanotubes promotes a great energy transfer from the incident beam to the culture medium, leading to ionization processes that give rise to secondary electrons.

The DEF results are shown in Figure 10b; it is important to note that the DEF values are different for the cytoplasm and the cell nucleus and when considering the whole cell lattice. For ZnTNT cell cytoplasm DEF = 1.31, cell nucleus DEF = 1.40 and for whole cell lattice DEF = 1.46, on the other hand, for NaTNT DEF = 1.21; 1.31; and 1.39, for cytoplasm, nucleus, and lattice, respectively. This difference is mainly due to the different concentrations of TNT internalized in the cell and in the nucleus region. In addition, the photoelectrons and the Auger electrons deposit their energy in the few micrometers in the vicinity of the TNTs.^69,124^ The slightly higher DEF values for ZnTNT compared to NaTNT occur because Zn has a higher atomic number than Na, and this means that the energy of the secondary electrons produced by ZnTNT has a slightly higher damage potential than that of NaTNT.

Finally, the DEF values for the matrix are slightly higher than the others, as they take into consideration the TNT clusters that were not internalized, and despite being to a lesser extent, they also indirectly lead to cell damage. These results reinforce the behavior observed by the experimental assays despite not reproducing the same cell survival fraction values. This is explained because this computational scenario is an approximation for the tests with U251 and U87 cells and because the quantities measured by the simulations describe the physical processes of the interaction of ionizing radiation with matter. Besides that, the processes subsequent to the physical processes, such as the creation of free radicals, are also very important, as they indirectly damage the DNA, which can lead to a decrease in the survival fraction of the cells.^51,124−126^

## 4 Conclusions

In this study, the synthesis of NaTNT and ZnTNT was achieved successfully. TEM observations indicated two potential routes for the internalization of nanotubes in GBM cells: endocytosis and diffusion. Our findings demonstrated favorable biocompatibility at the employed concentrations and exposure durations of ZnTNT, which is a relevant advantage in disease treatment. It was evident that TNTs could induce diverse radiosensitization effects across GBM cell lines, which might be attributed to their distinct resistance profiles. In totality, ZnTNT exhibited more promising outcomes when compared to NaTNT. Combining ZnTNT with ionizing radiation yielded reduced tumor proliferation, suppressed colony formation, and induced nucleus alterations in both GBM cell lines. Importantly, computational simulations using MCNP6.2 validated our experimental observations by confirming that incident beam energy transfer was greater in cases involving TNTs, substantiating the observed decreases in cell survival fractions. In conclusion, this study unveiled the promising potential of zinc-TNTs as a valuable tool in GBM RT treatment.

## Data Availability

The data used to plot Figures 4, 6, 7, and 8 are being submitted to the journal with the paper. However, the data are available from the corresponding author upon reasonable request subject to institutional data sharing policies.

## Supporting Information Available

Cellular experiments and table showing the average viability and number of Vero, U87MG and U251MG cells exposed to NaTNT and ZnTNT and dynamic cell population raw data from U87 and U251 cells exposed to NaTNT and ZnTNT with/without combination with irradiation (PDF)

## Abbreviations

GBM: glioblastoma
TNT: titanate nanotubes
RT: radiotherapy
ZnTNT: zinc-modified titanate nanotubes
NaTNT: sodium-modified titanate nanotubes
TEM: transmission electron microscopy
DEF: dose enhancement factor
EDS: energy-dispersive spectroscopy
STEM: scanning-transmission mode
FTIR: Fourier-transform infrared spectroscopy
ICP-MS: inductively coupled plasma mass spectrometry
XPS: X-ray photoelectron spectroscopy
MTT: 3-(4,5-dimethylthiazol-2-yl)-2,5-diphenyltetrazolium bromide solution, cytotoxicity assay
NMA: nuclear morphometric assay
MCM: Monte Carlo Method
