# Supplementary material for: Zinc-Modified Titanate Nanotubes as Radiosensitizers for Glioblastoma: Enhancing Radiotherapy Efficacy and Monte Carlo Simulations
Source: ACS Omega. 2024 Jun 28;9(27):29499–515. doi: 10.1021/acsomega.4c02125 (PMC11238320; doi:10.1021/acsomega.4c02125)
Supplement: Supplementary file 1 — ao4c02125_si_001.pdf [file ao4c02125_si_001.pdf]

Zinc-Modified Titanate Nanotubes as Radiosensitizers for Glioblastoma: Enhancing Radiotherapy Efficacy and Monte Carlo Simulations

Fernando Mendonça Diz[a,b‡], Wesley F. Monteiro[b‡], Iury Santos Silveira[c], Daniel Ruano[d,e], Eduardo Rosa Zotti[b], Rafael Diogo Weimer[b], Micael Nunes Melo[f], João Gabriel Schossler Lopes[g], Thamiris Becker Scheffel[a], Linda V. E. Caldas[c], Jaderson Costa da Costa[a], Fernanda Bueno Morrone[a,h], and Rosane Angélica Ligabue[b*]

[a] Preclinical Research Center, Brain Institute of Rio Grande do Sul, Pontifical Catholic University of Rio Grande do Sul - PUCRS, Porto Alegre/RS, Brazil.
[b] Graduate Program in Materials Engineering and Technology, Pontifical Catholic University of Rio Grande do Sul - PUCRS, Porto Alegre/RS, Brazil.
[c] Institute of Energy and Nuclear Research, National Nuclear Energy Commission – IPEN/CNEN. São Paulo/SP, Brazil.
[d] ALBA Syconhrotron Light Source, Cerdanuola del Vallès, Spain.
[e] Instituto de Tecnología Química, Universitat Politècnica de València-Consejo Superior de Investigaciones Científica (UPV-CSIC). Valencia, Spain.
[f] Institute of Technology and Research - ITP, Aracaju/SE, Brazil.
[g] Radiotherapy Service at Hospital São Lucas da Pontifical Catholic University of Rio Grande do Sul /Oncoclinic Group, Porto Alegre/RS, Brazil.
[h] School of Life and Health Sciences, Pontifical Catholic University of Rio Grande do Sul - PUCRS, Porto Alegre/RS, Brazil.

* Correspondence should be addressed to Rosane A. Ligabue: rligabue@pucrs.br

Table S1: Raw data of cellular experiments showing the average viability of Vero, U87MG, U21MG exposed to NaTNT and ZnTNT.

	Cell Viability - Vero					
NaTNT	Controle	5	15	25	50	100
	96,34	96,53	99,42	111,37	95,95	97,54
	97,69	103,28	113,87	105,78	97,30	104,17
	105,97	98,84	101,54	110,60	106,55	99,65
	100,00	99,28	92,97	120,10	91,35	100,39
	98,38	96,58	124,51	98,92	98,74	99,48
	101,62	105,05	94,96	107,57	93,87	105,05
	97,74	114,61	100,07	112,09	140,45	112,54
	100,26	102,00	95,22	100,65	98,71	105,21
	102,00	99,48	102,20	105,50	101,03	96,58
**Mean**	**100,00**	**101,74**	**102,75**	**108,06**	**102,66**	**102,29**
ZnTNT	Controle	5	15	25	50	100
	100,34	115,56	118,73	121,52	116,20	104,24
	97,67	108,33	114,42	121,14	120,13	104,21
	101,99	109,22	110,23	109,60	117,28	106,43
	99,93	98,95	101,67	114,06	115,49	106,67
	95,91	92,76	97,97	113,73	112,86	103,75
	104,16	93,52	94,39	93,85	112,44	99,91
	110,36	109,76	142,77	115,42	110,36	102,48
	92,77	102,89	108,68	115,06	114,10	104,39
	96,87	103,74	104,70	114,10	101,45	99,73
**Mean**	**100,00**	**103,86**	**110,39**	**113,16**	**113,37**	**103,53**

	Cell Viability - U87MG					Cell Viability - U251MG			
NaTNT	Controle	5	15	25	NaTNT	Controle	5	15	25
	110,06	98,81	84,45	90,851		114,59	125,57	126,91	131,995
	93,23	89,11	83,62	78,225		97,06	96,52	107,10	138,554
	96,71	73,10	73,10	70,997		88,35	88,35	113,25	116,198
	94,44	84,10	75,77	78,448		84,93	93,57	123,32	120,166
	104,79	102,01	99,04	75,192		103,21	91,08	116,01	129,307
	100,77	107,09	75,10	69,061		111,86	107,37	117,67	122,992
	99,37	101,18	82,92	83,093		81,00	93,94	90,53	107,011
	96,51	90,80	94,17	86,295		99,34	87,97	107,86	110,137
	104,13	101,18	100,84	90,104		119,66	118,10	111,56	112,695
	Cell Viability - U87MG					Cell Viability - U251MG			
ZnTNT	Controle	5	15	25	ZnTNT	Controle	5	15	25
	95,04	91,65	85,35	93,462		99,58	95,65	95,37	96,208
	98,79	90,68	94,79	92,978		97,61	92,98	96,07	105,478
	106,17	97,58	116,22	104,237		102,81	84,83	92,56	100,843
	111,54	117,01	126,84	139,621		101,24	97,24	98,81	106,244
	93,30	102,26	103,57	112,637		103,81	113,11	114,54	114,109
	95,16	102,80	100,51	99,636		94,95	95,81	106,24	97,521
	100,32	89,86	93,71	96,592		96,45	110,42	116,27	115,824
	103,81	99,60	115,72	90,457		98,10	101,25	98,55	107,411
	95,87	106,82	112,35	96,351		105,46	105,61	112,07	106,81

Table S2: Raw data of cellular experiments showing the average number of U87MG and U251MG cells exposed to NaTNT and ZnTNT.

	U251MG		
	**Control**	**NaTNT**	**ZnTNT**
Non-Irradiated	71,35922	96,60194	102,4272
	91,26214	94,17476	88,34952
	73,78641	92,23301	117,4757
	114,0777	75,24272	73,78641
	114,0777	142,233	111,165
	91,26214	148,0583	91,26214
	119,9029	146,1165	135,9223
	135,9223	142,7184	145,1456
	111,6505	153,8835	99,02913
	72,57281	94,41747	109,9515
	102,6699	84,70874	81,06796
	101,4563	150,9709	95,14563
Irradiated	102,427	45,534	44,66
	48,398	48,398	42,718
	76,699	46,117	62,621
	65,534	45,388	42,718
	51,456	48,058	48,398
	71,359	54,369	48,058
	87,379	48,058	45,388
	56,796	51,456	42,718
	65,049	45,388	56,796
	89,563	45,825	53,641
	56,966	46,893	42,718
	68,204	49,879	52,427

	U87MG		
	**Control**	**NaTNT**	**ZnTNT**
Non-Irradiated	93,40842	125,8695	131,8317
	103,3455	85,45876	129,1819
	92,08347	93,40842	126,532
	120,5697	120,5697	124,5445
	85,45876	108,6452	130,5068
	103,3455	79,49652	123,8821
	98,37695	105,6641	120,5697
	97,71448	89,43359	131,1693
	106,3266	106,9891	128,8506
	103,0142	114,6075	107,6515
	98,04571	97,54886	102,683
	104,836	93,2428	106,4922
	99,86751	116,4293	107,1547
	100,6956	91,50381	100,6956
	92,91156	81,31831	105,1673
Irradiated	9,937	7,751	11,659
	11,659	7,751	11,659
	15,568	19,41	12,587
	162,968	104,67	100,696
	198,079	89,434	81,484
	147,731	100,696	93,408
	180,523	97,052	91,09
	172,905	95,065	87,446
	164,127	98,874	92,249
	127,526	97,052	91,09
	130,341	96,058	89,268
	129,43	99,785	92,829
	138,87	96,555	90,179
	136,469	98,267	91,863
	126,146	96,997	90,262

Table S3: Dynamic cell population raw data from U87 and U251 cells exposed to NaTNT (5 µg.mL^{-1}) and ZnTNT (5 µg.mL^{-1}) with/without combination with irradiation (5Gy).

U251MG				
	Group			
Control	Non-Irradiated		Irradiated	
	Mean	SD	Mean	SD
N	94,10	0,40	84,00	1,1
SR	0,30	0,10	2,10	0,2
LR	3,40	0,30	9,50	1,2
NaTNT	Non-Irradiated		Irradiated	
	Mean	SD	Mean	SD
N	94,40	2,80	79,90	1,3
SR	1,00	0,30	1,10	0,4
LR	2,90	1,00	13,50	0,7
ZnTNT	Non-Irradiated		Irradiated	
	Mean	SD	Mean	SD
N	95,30	0,10	58,30	4,8
SR	0,20	0,00	6,50	4,4
LR	3,70	0,10	28,90	3,1

U87MG				
	Group			
Control	Non-Irradiated		Irradiated	
	Mean	SD	Mean	SD
N	89,28	0,92	79,32	4,56476
SR	3,02	0,63	2,27	1,96299
LR	3,88	1,26	9,56	1,31173
NaTNT	Non-Irradiated		Irradiated	
	Mean	SD	Mean	SD
N	90,10	1,30	75,60	3,83145
SR	6,50	1,20	15,47	0,61101
LR	1,40	0,30	1,87	1,61658
ZnTNT	Non-Irradiated		Irradiated	
	Mean	SD	Mean	SD
N	89,53	0,42	81,00	2,64575
SR	2,53	2,19	14,07	3,44287
LR	2,80	2,42	2,27	0,23094
